# CSA13 inhibits colitis-associated intestinal fibrosis via a formyl peptide receptor like-1 mediated HMG-CoA reductase pathway

**DOI:** 10.1038/s41598-017-16753-z

**Published:** 2017-11-27

**Authors:** Chunlan Xu, Sally Ghali, Jiani Wang, David Q. Shih, Christina Ortiz, Caroline C. Mussatto, Elaine C. Lee, Diana H. Tran, Jonathan P. Jacobs, Venu Lagishetty, Phillip Fleshner, Lori Robbins, Michelle Vu, Tressia C. Hing, Dermot P. B McGovern, Hon Wai Koon

**Affiliations:** 1 Center for Inflammatory Bowel Diseases, Vatche and Tamar Manoukian Division of Digestive Diseases, David Geffen School of Medicine at the University of California Los Angeles, Los Angeles, CA 90095 USA; 2 F. Widjaja Foundation, Inflammatory Bowel & Immunobiology Research Institute, Cedars-Sinai Medical Center, Los Angeles, CA 90048 USA; 3 Department of Gastroenterology, First Affiliated Hospital, China Medical University, Shenyang City, Liaoning Province China; 4 The Key Laboratory for Space Bioscience and Biotechnology, School of Life Science, Northwestern Polytechnical University, Xian, Shaanxi Province China

## Abstract

Many Crohn’s disease (CD) patients develop intestinal strictures, which are difficult to prevent and treat. Cationic steroid antimicrobial 13 (CSA13) shares cationic nature and antimicrobial function with antimicrobial peptide cathelicidin. As many functions of cathelicidin are mediated through formyl peptide receptor-like 1 (FPRL1), we hypothesize that CSA13 mediates anti-fibrogenic effects via FPRL1. Human intestinal biopsies were used in clinical data analysis. Chronic trinitrobenzene sulfonic acid (TNBS) colitis-associated intestinal fibrosis mouse model with the administration of CSA13 was used. Colonic FPRL1 mRNA expression was positively correlated with the histology scores of inflammatory bowel disease patients. In CD patients, colonic FPRL1 mRNA was positively correlated with intestinal stricture. CSA13 administration ameliorated intestinal fibrosis without influencing intestinal microbiota. Inhibition of FPRL1, but not suppression of intestinal microbiota, reversed these protective effects of CSA13. Metabolomic analysis indicated increased fecal mevalonate levels in the TNBS-treated mice, which were reduced by the CSA13 administration. CSA13 inhibited colonic HMG-CoA reductase activity in an FPRL1-dependent manner. Mevalonate reversed the anti-fibrogenic effect of CSA13. The increased colonic FPRL1 expression is associated with severe mucosal disease activity and intestinal stricture. CSA13 inhibits intestinal fibrosis via FPRL1-dependent modulation of HMG-CoA reductase pathway.

## Introduction

Inflammatory bowel diseases (IBD) such as ulcerative colitis (UC) and Crohn’s disease (CD) are complex autoimmune diseases associated with diverse disease presentations and variable responses to therapy^1^. In some cases, surgical resection of the intestine is necessary, which may negatively affect the patients’ quality of life. New therapeutic approaches are being actively sought after to improve therapeutic efficacy and the quality of medical treatment for IBD^2^.

N8 Medical, Inc. developed a non-peptide cationic steroid antimicrobial 13 (CSA13) as a gastrointestinal antibiotic. Administration of CSA13 was shown to reduce bacterial outgrowth in the abdomen of mice with peritoneal infection^3^. However, CSA13 has not yet been applied to treat gastrointestinal autoimmune diseases such as IBD. We hypothesize that CSA13 can treat colitis and colitis-associated intestinal fibrosis. To make CSA13 clinically applicable for this use, we collaborated with Southwest Research Institute (SWRI) in Texas to combine the CSA13 with the Eudragit polymer. This CSA13-Eudragit oral formulation releases CSA13 in alkaline pH, i.e., ileum and colon, ensuring that these target sites of intestinal fibrosis and colitis receive maximal levels of CSA13.

Formyl peptide receptor-like 1 (FPRL1) is an immunomodulatory mediator of cathelicidin^4^. CSA13 shares the cationic structure and antimicrobial function as natural cathelicidin^5^. FPRL1 is known to mediate the development of colitis in mouse models^6^. FPRL1-deficient mice developed milder colitis than wild-type mice during the inflammatory phase of DSS colitis, suggesting that FPRL1 mediates inflammation^6^. Interestingly, FPRL1-deficient mice developed more severe colitis than wild-type mice during the healing phase of DSS colitis^6^. Currently, the significance of colonic FPRL1 mRNA and protein expression in IBD patients (including CD patients with intestinal strictures) has never been reported in the literature.

Due to the importance of FPRL1 in intestinal inflammation, we hypothesize that colonic FPRL1 mRNA expression correlates with mucosal damages in IBD patients and stricture formation in CD patients. It is possible that FPRL1 is a drug target for anti-fibrogenic agents. We also hypothesize that CSA13 can interact with FPRL1 to mediate its anti-fibrogenic effect in colitis. This study includes a clinical data analysis of human colonic biopsies, fecal metabolomic analysis of mouse fecal samples, and functional studies to elucidate the anti-fibrogenic mechanism of CSA13.

## Results

### Colonic FPRL1 mRNA expression was positively correlated with histological damage in UC and CD patients

**Figure 1:**
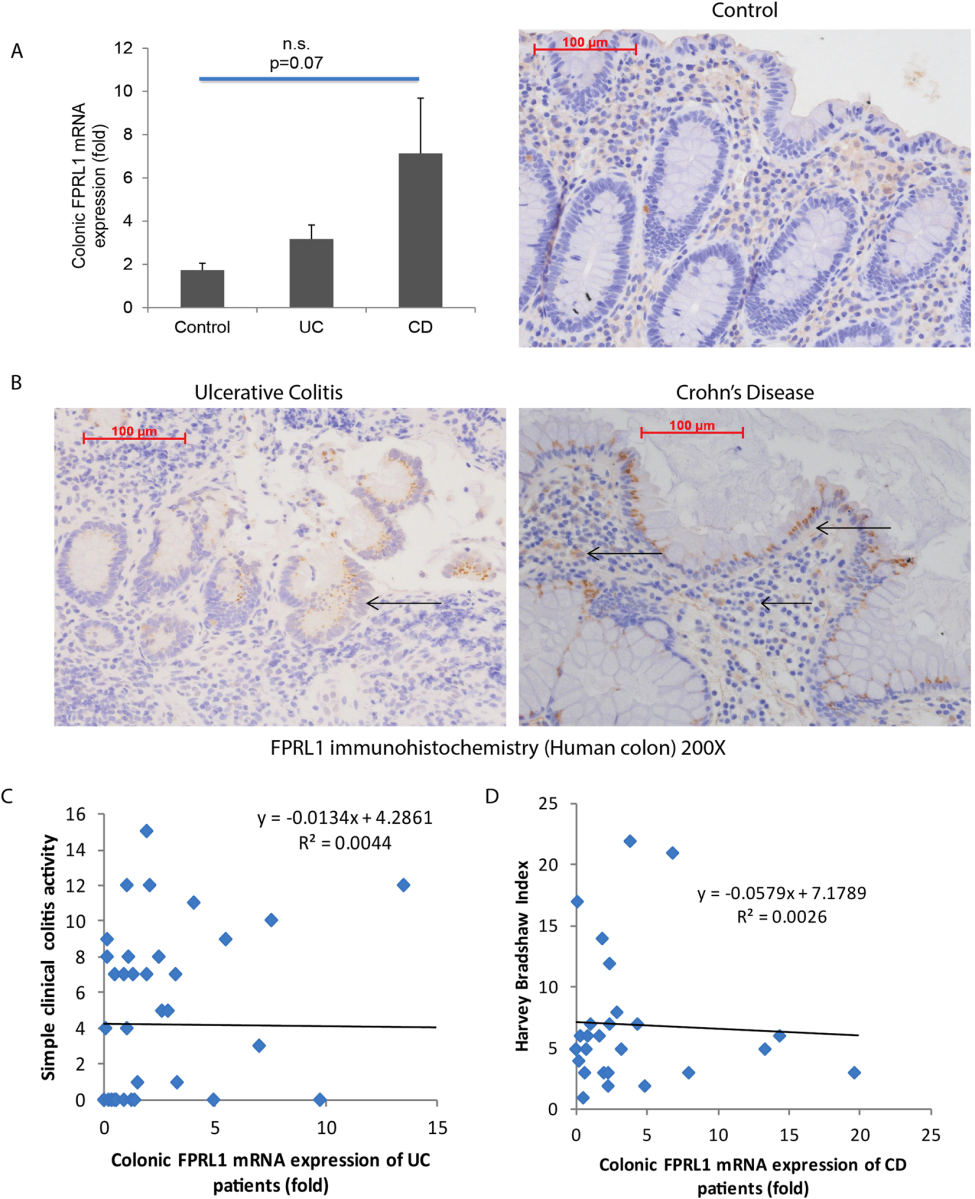
Colonic FPRL1 expression is present in IBD patients. (**A**) Colonic FPRL1 mRNA expression in normal, UC, and CD patients. (**B**) Immunohistochemistry of FPRL1 protein. The FPRL1 expression is located at the mucosal lining and the submucosal region as indicated by arrows. Magnification 200X. (**C**) Scatter plot shows that there was no correlation between colonic FPRL1 mRNA expression and clinical disease activity in UC patients. (**D**) Scatter plot shows that there was no correlation between colonic FPRL1 mRNA expression and clinical disease activity in CD patients. Data consist of colonic tissues from 40 normal, 50 UC, and 44 CD patients of cohort 1.

In cohort 1, there was no significant difference in colonic FPRL1 mRNA expression in both UC and CD patients, compared to the control group (Fig. 1A). We observed a similar trend of colonic FPRL1 mRNA expression in IBD patients from cohort 2 (Supplementary Figure 1A). FPRL1 immunohistochemistry showed that the protein expression of FPRL1 was located in the submucosal regions of all subjects (Fig. 1B). In some UC and CD patients, FPRL1 staining was found at the luminal side of the colonic epithelial cells (Fig. 1B).

**Figure 2:**
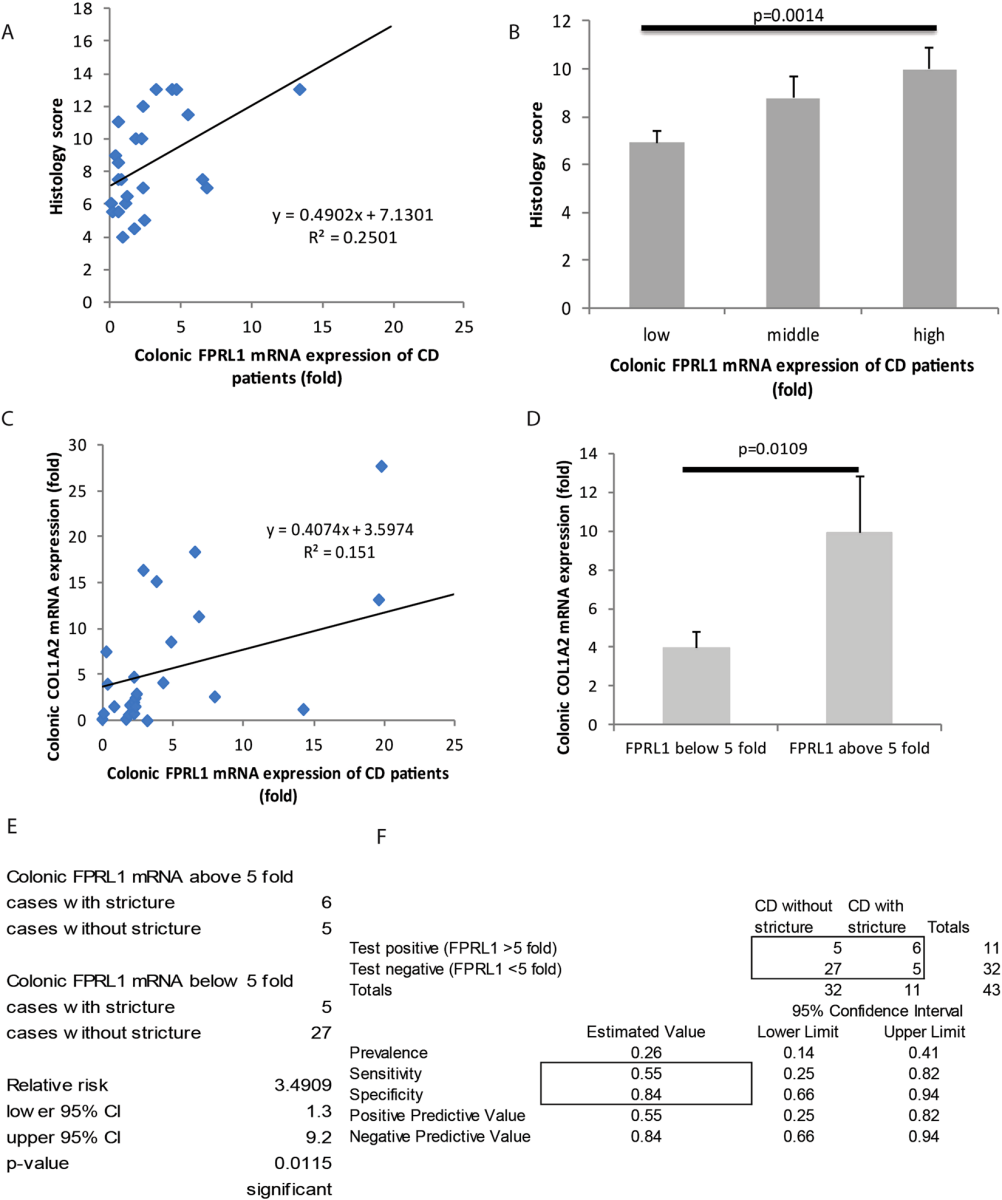
Colonic FPRL1 mRNA expression was positively correlated with histological damages and intestinal stricture of CD patients. (**A**) Scatter plot shows the positive correlation between colonic FPRL1 mRNA expression and histology score of CD patients. (**B**) The high colonic FPRL1 expression group had significantly higher histology score than the low colonic FPRL1 expression group. (**C**) Scatter plot shows the positive correlation between colonic FPRL1 mRNA expression and colonic collagen COL1A2 mRNA expression. (**D**) The high colonic FPRL1 expression group had significantly higher colonic collagen COL1A2 mRNA expression than the low colonic FPRL1 expression group. (**E**) The relative risk of colonic stricture in CD patients. (**F**) Sensitivity and specificity of colonic FPRL1 mRNA expression in indicating the presence of intestinal stricture in CD patients. Data consist of colonic tissues from CD patients of cohort 1.

In UC and CD patients, there was no correlation between colonic FPRL1 mRNA expression and clinical disease activity (Fig. 1C,D). However, increased colonic FPRL1 mRNA expression was directly proportional to histology score (Fig. 2A and Supplementary Figure 2A). The high tertile of colonic FPRL1 mRNA expression was associated with significantly increased histology score (Fig. 2B and Supplementary Figure 2B). The high colonic FPRL1 mRNA expression was associated with increased immune cell infiltration as reflected by the elevated immune cell subscore (Supplementary Table 2).

### CD patients with high colonic FPRL1 mRNA expression had an elevated relative risk of intestinal stricture

In cohort 1, colonic FPRL1 mRNA expression was positively correlated with colonic collagen COL1A2 mRNA expression (Fig. 2C). The CD patients with high colonic FPRL1 mRNA expression (above five fold) had significantly higher colonic COL1A2 mRNA expression than those with low colonic FPRL1 mRNA expression (below five fold) (Fig. 2D). This high FPRL1 population had a significantly higher relative risk of stricture than the low FPRL1 population (Fig. 2E). The high colonic FPRL1 mRNA expression had high specificity in indicating CD patients with stricture (Fig. 2F).

In cohort 2, the positive correlation between colonic FPRL1 and COL1A2 mRNA expression was also present (Supplementary Figure 1B,C). Both ileal and colonic FPRL1 mRNA expression are correlated with collagen mRNA expression (Supplementary Figure 1D). The correlation of colonic FPRL1 and collagen gene expression in CD disease cohorts 1 and 2 were similar. We decided not to combine the data from two cohorts since cohort 2 had no clinical data available.

### CSA13 inhibited colitis-associated intestinal fibrosis via FPRL1 in mice

**Figure 3:**
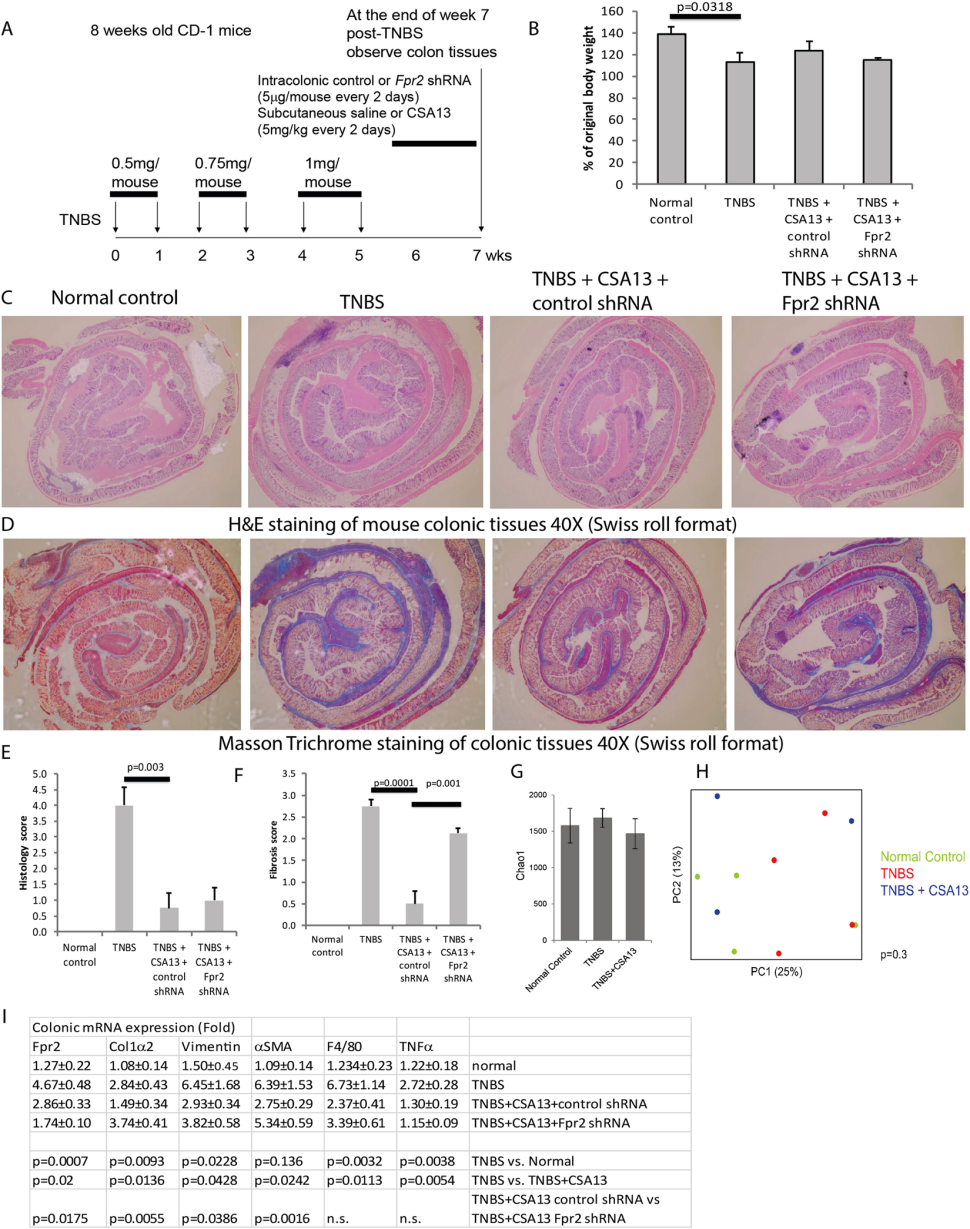
Subcutaneous CSA13 administration suppressed colonic fibrosis via FPRL1. (**A**) Experimental plan of TNBS colitis. (**B**) Changes in body weight. TNBS treatment significantly reduced body weight. (**C**) H&E staining. (**D**) Masson Trichrome staining. Blue color indicates deposition of collagen. (**E**) Histology score. (**F**) Fibrosis score. (**G**) Alpha diversity (richness as measured by Chao1) is shown for fecal samples of the mice (3–4 mice per group). (**H**) Principal coordinates plot of unweighted UniFrac for all mice. The significance of differences in microbial composition (beta diversity) across groups was determined using a permutational method (PERMANOVA) and the p-value is shown in the plot. (**I**) Colonic mRNA expression. Each group consists of 6 mice in 2 separate experiments.

To determine whether CSA13 can serve as an anti-fibrogenic drug, we subcutaneously administered CSA13 to the 2,4,6-Trinitrobenzenesulfonic acid (TNBS)-exposed mice with colonic fibrosis (Fig. 3A). TNBS treatment reduced body weight (Fig. 3B). The body weight loss of TNBS-treated mice was not significantly reversed after one week of subcutaneous CSA13 administration.

Chronic low-dose TNBS treatment led to mild colonic inflammation (Fig. 3C and E) with extensive deposition of collagen and fibrosis (Fig. 3D and F). One week of subcutaneous CSA13 administration significantly reduced histology score, colonic collagen deposition, and the fibrosis score in the TNBS-treated mice. *Fpr2* (mouse FPRL1 gene) shRNA treatment significantly reversed the CSA13-mediated inhibition of colonic fibrosis, but not inhibition of colonic histological damages (Fig. 3E,F). Based on these results, the anti-fibrogenic effects of CSA13 appear to be mediated by FPRL1.

We analyzed the fecal microbiota using next-generation 16 S sequencing on Illumina platform. Neither TNBS nor CSA13 treatment affected microbial richness, evenness, and phylogenetic diversity (Fig. 3G). No significant overall change in microbial community composition was seen by unweighted UniFrac analysis (Fig. 3H). CSA13 was not associated with modulation of gut microbiota.

CSA13 treatment significantly inhibited colonic collagen *Col1a2* mRNA expression and fibroblast accumulation (*αSMA* and vimentin) in the mice with TNBS colitis that was reversed by *Fpr2* shRNA (Fig. 3I). Colonic *Fpr2* mRNA expression was significantly reduced by *Fpr2* shRNA. CSA13 also inhibited macrophage accumulation (*F4/80*) and cytokine expression (*Tnf*); however, this inhibition was not affected by the *Fpr2* shRNA. FPRL1 specifically mediated the anti-fibrogenic effect of CSA13.

### Anti-fibrogenic effect of oral CSA13-Eudragit was not dependent on intestinal microbiota

**Figure 4:**
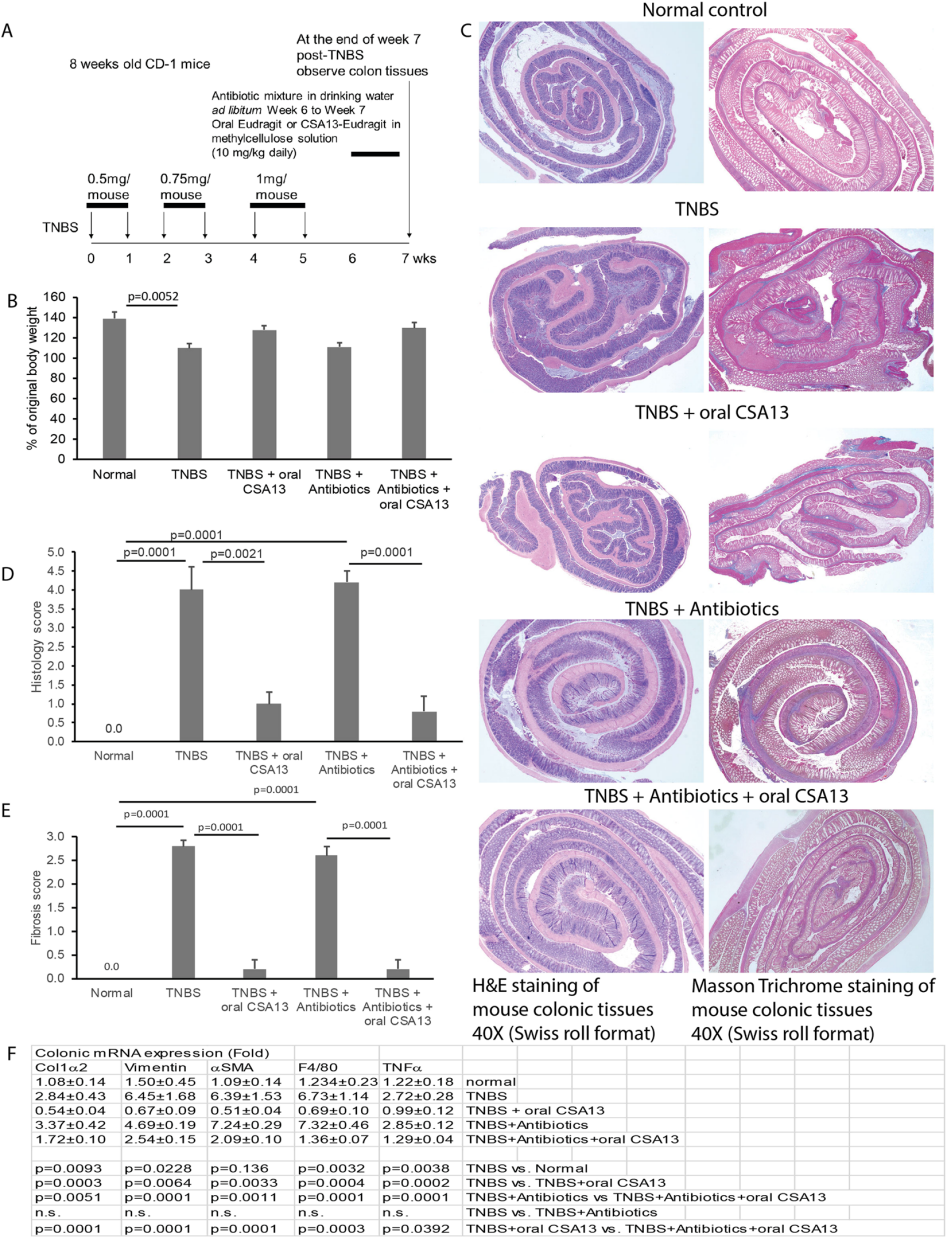
The anti-fibrogenic effect of the oral CSA13-Eudragit was not affected by antibiotic treatment. (**A**) Experimental plan of TNBS colitis. (**B**) Changes in body weight. TNBS treatment significantly reduced body weight. (**C**) H&E staining (left) and Masson Trichrome staining (right). Blue color indicates deposition of collagen. (**D**) Histology score. (**E**) Fibrosis score. (**F**) Colonic mRNA expression. Each group consists of 6 mice in 2 separate experiments.

To evaluate whether the oral CSA13-Eudragit is also effective against intestinal fibrosis, we administered CSA13-Eudragit to mice with chronic TNBS colitis (Fig. 4A). Oral CSA13-Eudragit administration significantly ameliorated the colonic damage and fibrosis in the TNBS-exposed mice (Fig. 4C–E). Similar to Fig. 3, short-term one-week administration of oral CSA13-Eudragit did not significantly alter the TNBS-mediated body weight loss (Fig. 4B).

To determine whether microbiota is involved in the development of colonic fibrosis and the anti-fibrogenic mechanism of CSA13, we administered a mixture of antibiotics to suppress microbiota in the fibrogenic stage, i.e., the last week of the experiment. This antibiotic regimen was used to suppress intestinal microbial alpha diversity (Chao1 data not shown) and facilitate *C. difficile* infection in our previous *C. difficile* infection study^7^. Interestingly, antibiotics not only had no effect to the TNBS-mediated colonic inflammation and fibrosis (Fig. 4D,E), but also did not influence colonic collagen, vimentin, or *αSMA* mRNA expression in TNBS-treated mice (Fig. 4F). Although antibiotic treatment moderately reversed the CSA13-mediated suppression of fibrogenic and inflammatory gene expression (Fig. 4F), the histology and fibrosis scores of the CSA13-treated mice with or without antibiotic treatment remained low (Fig. 4D,E). Thus, the intestinal microbiota is not an essential mechanism of action in the anti-fibrogenic effect of CSA13.

### CSA13 inhibits colonic 3-hydroxy-3-methyglutaryl-coenzyme A reductase (HMG-CoA reductase)-mediated fibroblast accumulation

**Figure 5:**
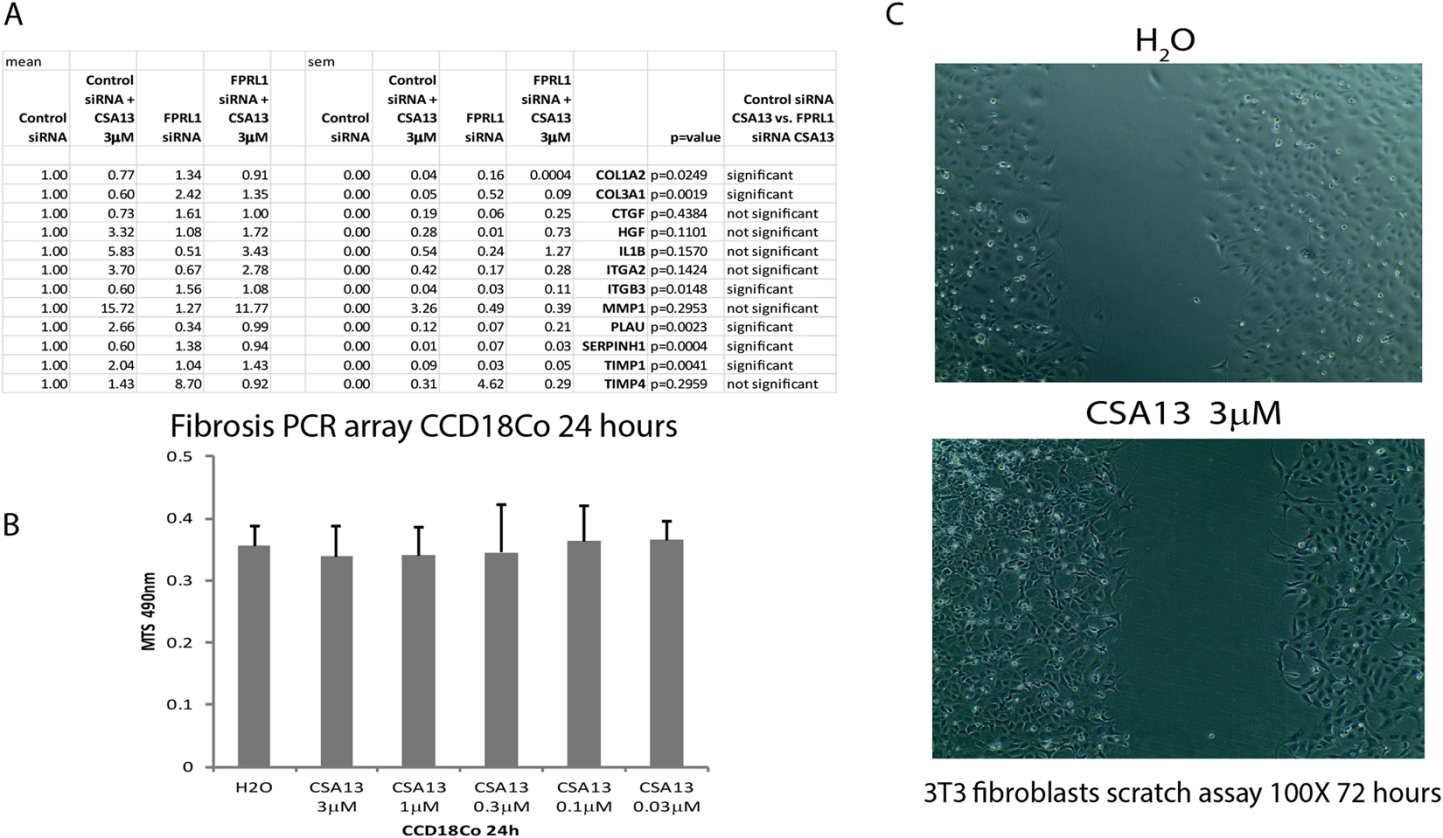
CSA13 did not affect cell viability and cell migration of fibroblasts. (**A**) Human CCD18Co fibroblasts were transiently transfected with control or FPRL1 siRNA overnight, followed by CSA13 for 24 hours. Each PCR array (Human Fibrosis PAHS120ZE-4, Qiagen, CA) detects 84 genes. Only CSA13-dependent genes are shown here. (**B**) The CCD-18Co fibroblasts were treated with CSA13 for 24 hours. The cell viability was determined by MTS assay at 490 nm. (**C**) The 3T3 fibroblasts were treated with CSA13 for 72 hours. The scratch gaps were recorded at 100X magnification. The results are representative of 3 experiments.

In cultured human colonic fibroblasts, CSA13 mildly inhibited collagen COL1A2 mRNA expression in an FPRL1-dependent manner (Fig. 5A). Cell viability and cell migration of fibroblasts were not affected by CSA13 (Fig. 5B,C). As cell culture model was not able to address how CSA13 inhibited fibroblast accumulation, we believe that CSA13 may change the intestinal chemical environment leading to inhibition of fibroblast accumulation.

**Figure 6:**
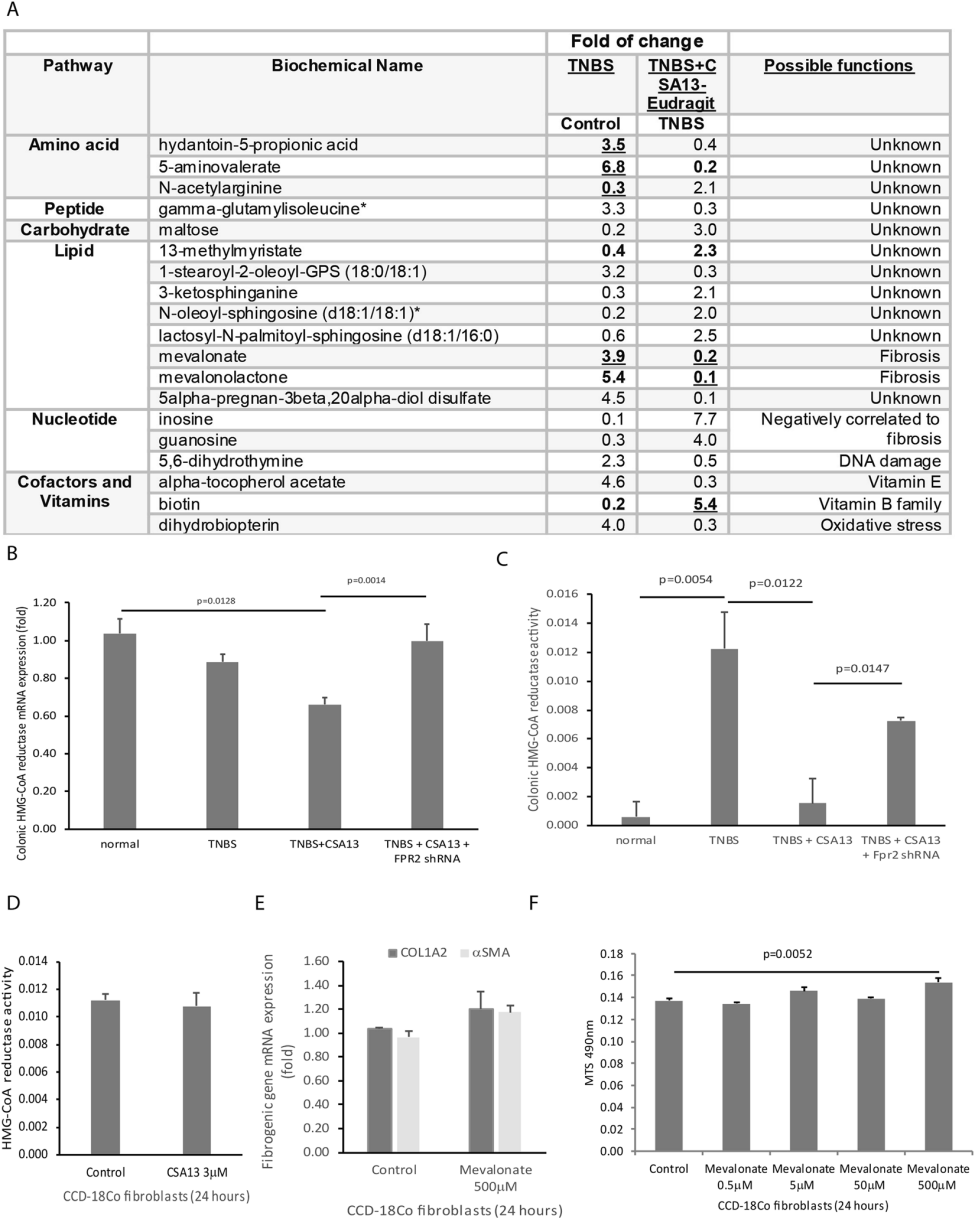
Fecal metabolomic analysis revealed the relevance of HMG-CoA reductase pathway. (**A**) Fecal samples were collected at the end of the experiments. Fecal metabolomic analysis showed the metabolites changed by TNBS and oral CSA13-Eudragit exposure. Only statistically-changed metabolites were shown here. (**B**) Colonic HMG-CoA reductase mRNA expression. (**C**) Colonic HMG-CoA reductase activity. 6 mice per group. (**D**) HMG-CoA reductase activity in human colonic fibroblasts after incubation with CSA13 for 24 hours. (**E**) Collagen mRNA expression in human colonic fibroblasts after incubation with mevalonate for 24 hours. (**F**) The CCD18Co fibroblasts were incubated with mevalonate for 24 hours. The cell viability was determined by MTS assay at 490 nm. The results are representative of three experiments.

Metabolomic analysis of fecal samples indicated that 19 out of 790 detected fecal metabolites were significantly influenced by both TNBS and oral CSA13-Eudragit (Fig. 6A). Fecal mevalonate levels were significantly increased in TNBS-treated mice, which was suppressed by oral CSA13-Eudragit administration (Fig. 6A). Mevalonate is the product of HMG-CoA reductase pathway for cholesterol synthesis^8^ and intestinal fibrosis^9^. Inhibition of HMG-CoA reductase with statin ameliorates intestinal inflammation in TNBS-treated rats^10^ and causes fibroblast apoptosis^11^.

TNBS exposure significantly increased colonic HMG-CoA reductase activity without altering the colonic HMG-CoA reductase mRNA expression (Fig. 6B,C). Oral CSA13-Eudragit significantly reduced colonic HMG-CoA reductase mRNA expression and activity and was reversed by *Fpr2* shRNA (Fig. 6B,C), suggesting that CSA13-mediated inhibition of HMG-CoA reductase expression and activity is FPRL1 dependent.

**Figure 7:**
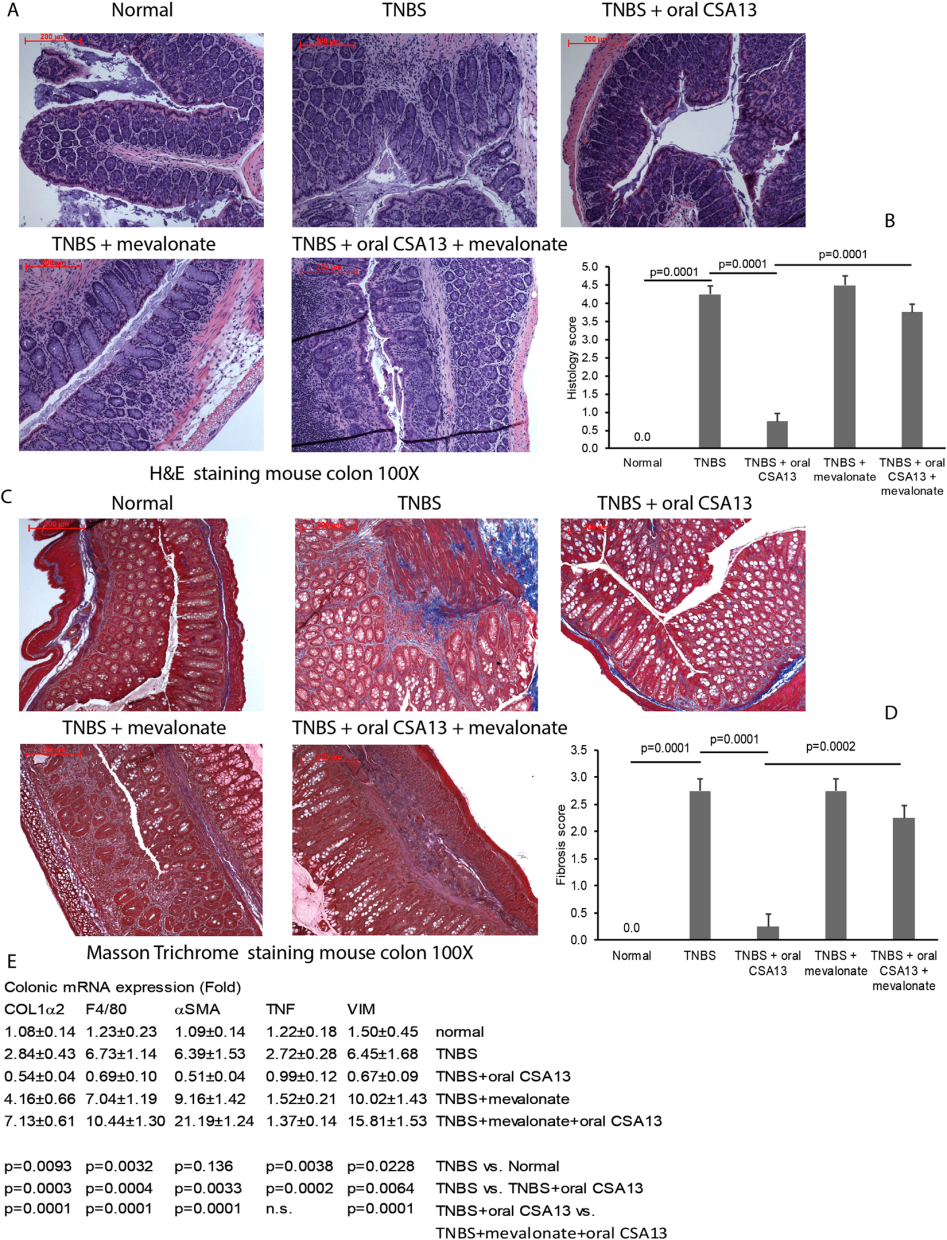
Intracolonic mevalonate administration reversed the anti-fibrogenic effect of CSA13 in TNBS-treated mice. TNBS-treated mice were treated with oral-Eudragit (10 mg/kg) and/or intracolonic mevalonate (10 mg/kg) daily during the last week of experiment. (**A**) H&E staining. (**B**) Masson Trichrome staining. (**C**) Histology score. (**D**) Fibrosis score. (**E**) Colonic mRNA expression. 6 mice per group.

HMG-CoA reductase activity in cultured colonic fibroblasts was not affected by CSA13 exposure (Fig. 6D). Mevalonate moderately promoted fibroblast cell proliferation without altering collagen expression and myofibroblast activation (Fig. 6E,F). Intracolonic mevalonate administration (50 mg/kg) during weeks 6–7 did not exacerbate the colonic histological damages and fibrosis in the TNBS-exposed mice but did reverse the anti-inflammatory and anti-fibrogenic effects of oral CSA13-Eudragit treatment (Fig. 7A–D). The increase of fibroblast marker (vimentin and αSMA) mRNA expression suggested an accumulation of fibroblasts upon mevalonate treatment (Fig. 7E).

## Discussion

The biological significance of FPRL1 in IBD patients may be bidirectional due to its pro-inflammatory and anti-inflammatory functions at different stages of colitis^6^. FPRL1 is expressed in colonic epithelial cells^12^, immune cells^13^, and fibroblasts^14^. In addition, the diverse ligands of FPRL1 may mediate different signaling pathways and physiological effects^15^. It is difficult to conclude whether the increased colonic FPRL1 mRNA expression is protective or detrimental to the IBD patients. However, the FPRL1 in the intestinal tissues may be a novel drug target for IBD therapy.

Our findings show that the anti-fibrogenic effects of CSA13 are mediated via an FPRL1-dependent metabolic pathway, rather than through antimicrobial function. Fecal mevalonate levels were consistent with colonic HMG-CoA reductase activity (Fig. 6A and C). We do not know the source of fecal mevalonate because many kinds of cells express HMG-CoA reductase and produce mevalonate. Although in-depth determination of cellular mevalonate synthesis is beyond our scope of investigation, mevalonate-mediated fibroblast cell proliferation is important in fibrogenesis (Fig. 6F). The relevance of metabolites in intestinal fibrosis other than mevalonate is undetermined. In future research, we will continue to explore whether other metabolites are involved in the development of intestinal fibrosis.

FPRL1 expression can be found in colonic epithelial cells (Fig. 1B) and immune cells^13^. The high FPRL1 tertile group had a significantly higher immune cell subscore than the low FPRL1 tertile group in IBD patients (Supplementary Table 2). The mucosal infiltration of FPRL1-expressing immune cells may be associated with severe histological damages (Supplementary Table 2).

The Crohn’s disease patients with colonic FPRL1 mRNA expression above 5 fold had a higher relative risk of intestinal stricture than those below 5 fold (Fig. 2E,F). Both groups above and below 5 fold had no significant difference in histology score and immune cell subscore (Supplementary Table 3, upper panel) because some of the CD patients in the low FPRL1 group (below 5 fold) belonged to the high FPRL1 tertile group (above 4.34 fold), which contained a high histology score and immune cell subscore (Supplementary Table 2). Increasing colonic FPRL1 mRNA expression beyond five fold was no longer associated with additional histological damage and immune cell infiltration. The occurrence of stricture had no correlation with histology score and immune cell subscore (Supplementary Table 3, lower panel).

CSA13 has advantages over natural cathelicidin due to chemical stability^16^. Natural cathelicidin (LL-37) is unsafe in systemic use since it causes significant hemolysis of human red blood cells^17^. Exposure of red blood cells to CSA13 up to 12 μM does not cause hemolysis^18^. An N8 Medical-sponsored study demonstrated that daily administration of oral CSA13-Eudragit up to 200 mg/kg for seven days did not cause mortality or liver and kidney (ALT, AST, creatinine) toxicity in rats (confidential corporate data not shown). CSA13, unlike cathelicidin, has a high safety margin for clinical application.

Both routes of CSA13 administration demonstrated effective suppression of colitis and fibrosis. The drug release profile of the CSA13-Eudragit has been extensively studied by N8 Medical, Inc. and SWRI (confidential corporate data not shown). The Eudragit polymer has been used to make the oral formulation of mesalamine (Asacol) that induced and maintained remission in IBD patients effectively and safely^19^. FPRL1 mRNA and protein expression are present in the intestine of IBD patients. Oral CSA13-Eudragit administration may be the best approach to maximize exposure of intestinal FPRL1 as this formulation releases CSA13 in ileum and colon.

Inhibition of colonic FPRL1 with intracolonic administration of *Fpr2* shRNA moderately increased colonic collagen Col1a2 mRNA expression, but did not worsen colonic histological damages and fibrosis in the TNBS-treated mice (data not shown). We speculate that the intestinal inflammation and fibrosis was at the threshold level and left little room for exacerbation after *Fpr2* inhibition.

Inhibition of FPRL1 by transient siRNA transfection increased collagen mRNA expression and reversed the anti-fibrogenic effects of CSA13 treatment in human colonic fibroblasts (Fig. 5A). Four FPRL1 agonists (WKYMVM, WKYMVm, MMK1, TC-FPR43, each at 10μM) significantly reduced collagen mRNA expression in cultured human colonic CCD18Co fibroblasts respectively (data not shown). Based on our evidence, CSA13 may be an FPRL1 pathway activator.

Fluorescent BODIPY-conjugated CSA13 binding to human colonic fibroblasts was not significantly affected by pretreatment of FPRL1 inhibitor WRW4 (data not shown), suggesting that CSA13 did not bind to FPRL1 receptor directly. We speculate that CSA13 may bind to another cell surface target and then mediate its downstream effects via FPRL1. The discovery of CSA13-binding targets is interesting, but is beyond the scope of this study.

CSA13 was not able to inhibit lipopolysaccharide-induced pro-inflammatory gene mRNA expression in macrophages (Supplementary Figure 3A). However, low concentration of CSA13 promoted cell migration without affecting cell viability of human colonic epithelial cells (Supplementary Figure 3B,C), suggesting that CSA13 may ameliorate mucosal damages by promoting epithelial healing process.

The 16 S sequencing of mouse fecal samples demonstrated that there was no significant change in the microbial environment during the fibrogenic phase of chronic TNBS colitis (Fig. 3G,H). Antibiotics are effective in suppressing acute DSS and TNBS colitis^20,21^, but they failed to influence the course of chronic DSS colitis^21^. The failure of antibiotics in altering the development of colonic inflammation and fibrosis suggested the lack of microbiota involvement (Fig. 4). CSA13 is unlikely to mediate its anti-fibrogenic effects via microbiota in the TNBS-exposed mice.

We cannot exclude this possibility in other animal models of intestinal fibrosis. In a small cohort of animals, CSA13 inhibited colitis with significantly increased colonic *Akkermansia* abundance in the mice with T-cell colitis (Supplementary Figure 4). *Akkermansia* is associated with anti-inflammatory effects in colitis^22^. The potential involvement of microbiota in the CSA13 treatment with other animal models of colitis should be examined in the future.

In conclusion, colonic FPRL1 expression is associated with colonic histological damage in IBD patients and presence of stricture in CD patients. CSA13 represents a new class of anti-fibrogenic drug, which inhibits HMG-CoA reductase-mediated mevalonate production, reduce fibroblast accumulation, and ameliorate intestinal fibrosis via FPRL1 activation.

## Methods

### Cohort 1

Human colonic biopsies were obtained from Cedars-Sinai Medical Center after informed consent. Inclusion criteria: Involved UC and CD colonic biopsies (IBD groups) or non-IBD colonic biopsies (control groups) from surgical resection from male or female patients, who were able to make informed consent independently, were included. Control group samples were obtained from non-IBD patients with colorectal cancer/polyp, diverticulitis or colon mass. Exclusion criteria: No pregnant women, prisoners, or minors under age 18 were included. Baseline characteristics were outlined in Supplementary Table 1–3.

Histological damage was blindly assessed by two investigators who scored the H&E stained specimens as described previously^23^. The scoring system assesses various features of acute and chronic intestinal inflammation in the mucosa of CD patients including (A) epithelial damage (0–2), (B) architectural change (0–2), (C) infiltration of mononuclear cells in the lamina propria (0–2), (D) infiltration of polymorphonuclear cells in the lamina propria (0–2), (E) presence of polymorphonuclear cells in epithelium (0–2), (F) presence of erosion and ulcers (0–1), and (G) presences of granuloma (0–1). The histology score is the sum of the above-mentioned parameters. Immune cell subscore is the sum of C, D, and E. We obtained one biopsy specimen from the involved region of intestine per patient.

Simple clinical colitis activity index was used to assess the clinical disease activity of UC patients in cohort 1^24^. Harvey Bradshaw Index was used to assess the clinical disease activity of CD patients in cohort 1^25^. The diagnosis of strictures was determined by imaging procedures (CT scans, MRI, and transabdominal ultrasonography) or colonoscopy.

### Cohort 2

TissueScan® Crohn’s and Colitis tissue cDNA array plates (#CCRT101 and #CCRT102) of colon and ileum were purchased from Origene Inc. PCR was performed after addition of master mix and primers as described previously^26^. However, histology score, disease activity indices, medications, and clinical data, except for tissue location, age, and gender of this cohort, are unavailable. Baseline characteristics were outlined in Supplementary Table 4.

### Immunohistochemistry of FPRL1

Immunohistochemistry was performed by Translational Pathology Core Laboratory (TPCL) of UCLA. Colonic biopsies were fixed in 4% paraformaldehyde and embedded in paraffin. Sections were incubated with a rabbit polyclonal anti-FPRL1 antibody (ab63023, Abcam) overnight at 4 °C (1:600 dilution) as described previously^27^. Images were taken with a Zeiss AX10 microscope.

### Production of CSA13-Eudragit

The CSA13 was coated with Eudragit FS30D polymer. This pH-responsive polymer is insoluble in an acidic environment but dissolves in mildly alkaline environment (i.e., pH 7 or above), which is ideal for colonic delivery. CSA13-Eudragit was packaged into microparticles using an SWRI-patented spinning disk atomization technology. This coating technology prevented leakage of CSA13 in acidic, aqueous solution. The CSA13-Eudragit microparticles (0.2–0.3 mm diameter) were dried into free-flow powder. The CSA13-Eudragit was suspended in 0.5% methylcellulose solution.

### TNBS chronic colitis-associated intestinal fibrosis model and T-cell transfer colitis model

8–10 weeks old female CD-1 mice (stock number #022) were purchased from Charles River Laboratories and maintained in the animal facility at the University of California Los Angeles under standard environmental conditions. Mice were injected with TNBS solution or 30% ethanol (50 μL) via enema weekly for 5 weeks as described previously^28^. After the last TNBS injection, the mice were held for two additional weeks to develop colonic fibrosis.

8 weeks old female c57BL/6 donor mice (stock number 000664) were purchased from Jackson Laboratories. CD4 + CD45Rb^hi^ T-cells were prepared and injected into the female RAG^−/−^ mice (stock number 002216), following a previously published protocol^29^. Colitis developed at ten weeks after T-cell injection.

(Subcutaneous CSA13 treatment and microbiome/metabolomic analysis) Mice were injected with CSA13 in saline (100 μL) under the skin daily during the weeks 7–8. Inhibition of FPRL1 was achieved by intracolonic *Fpr2* shRNA administration via InvivoJet transfection reagent during days 6–10 as described previously^30^. The mice with T-cell transfer colitis were injected with CSA13 subcutaneously between weeks 9–10.

At the end of the experiments, fecal samples were collected for 16 S sequencing (UCLA Microbiome Center). DNA extraction, amplification of the V4 region of 16 S ribosomal RNA gene, and 2 × 150 bp sequencing on an Illumina HiSeq. 2500 were performed as previously described^31^. Observable taxonomic units were picked against the May 2013 version of the Greengenes database, pre-filtered at 97% identity, in QIIME v1.9.1^32^. Sequence depth ranged from 166,423 to 406,785. Alpha diversity metrics (i.e., bacterial diversity within a sample) and beta diversity (differences in composition across samples) were calculated using data rarefied to 166,423 sequences. Alpha diversity metrics included Faith’s phylogenetic diversity metric, Chao1, and Shannon index. Beta diversity was calculated using unweighted UniFrac and visualized by principal coordinates analysis. Adonis, a permutational analysis of variance, was performed using 100,000 permutations to test for differences in beta diversity across the groups^33^.

(Oral CSA13-Eudragit administration) Mice were fed with the CSA13-Eudragit in 0.5% methylcellulose suspension (100μL) daily during weeks 7–8. Mice in the antibiotic treatment groups were provided with the antibiotic mixture (kanamycin 40 mg/kg, gentamicin 3.5 mg/kg, colistin 4.2 mg/kg, metronidazole 21.5 mg/kg, and vancomycin 4.5 mg/kg) in water *ad libitum* during weeks 7–8 as described previously^7^. Fecal samples were used for metabolomic analysis (Metabolon, Inc.).

### Histological evaluation of mouse colonic samples

Colonic tissues were fixed, sectioned and stained with H&E. Microphotographs were recorded at multiple locations and blindly scored by two investigators as described previously^28^. Masson Trichrome staining and fibrosis scoring were performed as described previously^28^.

### Cell Cultures

Human CCD-18Co colonic fibroblasts (2 × 10^6^ cells/plate) were cultured in minimal essential medium Eagle’s medium (ATCC, Manassas, VA) containing 10% fetal bovine serum (Invitrogen) and 1% penicillin/streptomycin (Invitrogen)^28^. Mouse RAW264.7 macrophages (2 × 10^6^ cells/plate) were cultured in DMEM containing 10% fetal bovine serum and 1% penicillin/streptomycin^7^. Mouse 3T3 fibroblast (2 × 10^6^ cells/plate) were cultured in DMEM containing 10% calf and 1% penicillin/streptomycin. Human colonic epithelial NCM460 cells (2 × 10^6^ cells/plate) were cultured in M3D containing 10% fetal bovine serum and 1% penicillin/streptomycin^30^. Cells were serum starved overnight before experiments.

### Cell viability assay and HMG-CoA reductase activity assay

Serum-starved NCM460 colonic epithelial cells and CCD-18Co fibroblasts were seeded in 96-well plates (1 million cells/plate) and were exposed to CSA13 or water (vehicle control) for 24 hours. 20 μl of CellTiter AQeuous One solution (MTS tetrazolium compound, Promega) was added to each well and incubated at 37 °C for 30 minutes. Absorbance at 490 nm (indicating cell viability) was measured using a 96-well plate reader as described previously^34^. The HMG-CoA reductase activity assays (ab204701, Abcam) were performed according to manufacturer’s instructions.

### Quantitative real-time RT-PCR and PCR arrays

Total RNA was isolated by an RNeasy kit (#74104, Qiagen, Valencia, CA) and reverse transcribed into cDNA by a Superscript III kit (#11752, Invitrogen, CA). Quantitative PCR reactions were run with Fast Universal PCR master mix (#4352042, Invitrogen) in an ABI Prism 7700 Fast sequence detector System^27^. The mRNA expression was determined by using cataloged primers (Invitrogen) for human *FPRL1* (Hs02759175_s1) and collagen COL1A2 (Hs01028956_m1).

For PCR arrays, RNA was converted to cDNA with RT^2^ First Strand kit (#330401, Qiagen). Mouse antibacterial response PCR arrays (PAMM-148Z) and human fibrosis PCR arrays (PAHS-120Z) were performed with RT^2^ SYBR Green PCR master mix (#330501, Qiagen) in Bio-Rad CFX384 PCR machine. All gene expression analyses were normalized to 18 S (Hs99999901_s1). Results were expressed as relative fold difference.

### Statistical analyses

Colonic and ileal FPRL1 mRNA expression were arranged in low-to-high order. The entire range of data was divided into three equal tertiles ($1/3$, $1/3$, $1/3$), with cut-off points denoted between the three tertiles. High FPRL1 mRNA expression was correlated with severe mucosal disease activity and high rate of stricture. The n-number of each tertile is shown in Supplementary Table 1–4.

Results were expressed as mean +/− SEM, unless specified otherwise. Bar graphs and scatter plots with R^2^ values were made using Microsoft Excel software program. Data were analyzed by using the Prism professional statistics software program (GraphPad, San Diego, CA). Unpaired Student’s t-tests were used for intergroup comparisons. Relative risk, sensitivity, specificity, positive predictive value (PPV), and negative predictive values (NPV), with 95% confidence intervals (CI), were calculated using a clinical calculator website: http://vassarstats.net/clin1.html. The p values are shown in each figure.

### Ethics Statement

All experimental protocols of the cohort 1 study were approved by institutional review boards (Cedars-Sinai Institutional Review Board, IRBs 3358 and 23705, and UCLA Institutional Review Board IRB-11–001527). All samples were collected during the indicated diagnostic procedure between 2010–2014. Informed consent was obtained from all subjects by the Cedars-Sinai Medical Center. Separate informed consent was waived by UCLA IRB. All methods were carried out in accordance with relevant guidelines and regulations.

Cohort 2 from Origene, Inc. did not have personally identifiable information. All samples were collected under IRB approved protocols. Informed consent was obtained from all subjects. Separate IRB and informed consent were waived by UCLA IRB. All methods were carried out in accordance with relevant guidelines and regulations.

Animal studies were approved by the Institutional Animal Research Committee of UCLA (#2007–116). All methods were carried out in accordance with relevant guidelines and regulations.

### Data Availability Statement

The data and materials used in this study are available at Dr. Koon’s laboratory.

## Electronic supplementary material

Supplementary Information
