# Supplementary material for: CSA13 inhibits colitis-associated intestinal fibrosis via a formyl peptide receptor like-1 mediated HMG-CoA reductase pathway
Source: Sci Rep. 2017 Nov 27;7:16351. doi: 10.1038/s41598-017-16753-z (PMC5703874; doi:10.1038/s41598-017-16753-z)

CSA13 inhibits colitis-associated intestinal fibrosis via a formyl peptide receptor like-1 mediated HMG-CoA reductase pathway.

[1, 4]Chunlan Xu, [1]Sally Ghali, [1,3]Jiani Wang, [2]David Q. Shih, [1]Christina Ortiz, [1]Caroline C. Mussatto, [1]Elaine C. Lee, [1]Diana H. Tran, [1]Jonathan P. Jacobs, [1]Venu Lagishetty, [2]Phillip Fleshner, [2]Lori Robbins, [2]Michelle Vu, [2]Tressia C. Hing, [2]Dermot PB McGovern, [1]Hon Wai Koon*

Supplementary Info File

Supplementary Table and Figure Legends:

Supplementary Table 1

Baseline characteristics of cohort 1, sorted by disease group.

Supplementary Table 2

Baseline characteristics of cohort 1, sorted by intestinal FPRL1 mRNA expression. NS indicated no statistically significant differences between groups.

Supplementary Table 3

(Upper table) Baseline characteristics of cohort 1 Crohn's disease patients, sorted by intestinal FPRL1 mRNA expression. (Lower table) Baseline characteristics of cohort 1 Crohn's disease patients, sorted by the presence of intestinal stricture. NS indicated no statistically significant differences between groups.

Supplementary Table 4

Baseline characteristics of cohort 2. NS indicated no statistically significant differences between groups.

Supplementary Figure 1

Ileal and colonic FPRL1 mRNA expression are associated with intestinal collagen expression.

(A) Intestinal FPRL1 mRNA expression in control, UC, and CD patients. Both UC and CD patients had increased intestinal FPRL1 mRNA expression, compared to normal control group. The difference was not statistically significant. (B) Scatter plot shows the positive correlation between intestinal FPRL1 mRNA expression and intestinal collagen COL1A2 mRNA expression. (C) The high intestinal FPRL1 expression group had significantly higher intestinal collagen COL1A2 mRNA expression than the low intestinal FPRL1 expression group. (D) Scatter plot shows the positive correlation between ileal and colonic FPRL1 mRNA expression and their respective collagen mRNA expression. Data consist of 17 colonic and 18 ileal samples of cohort 2.

Supplementary Figure 2

Colonic FPRL1 mRNA expression is positively correlated with histological damages in UC patients.

(A) Scatter plot shows the positive correlation between colonic FPRL1 mRNA expression and histology score. (B) The high colonic FPRL1 expression group had significantly higher histology score than the low colonic FPRL1 expression group. Data consist of colonic tissues from IBD patients of cohort 1.

Supplementary Figure 3

CSA13 promoted epithelial cell migration.

(A) Serum-starved mouse Raw264.7 macrophages were treated with LPS (1 μg/ml) and/or CSA13 (3μM) for 4 hours. The mRNA expression was determined by mouse antibacterial response PCR arrays. LPS increased pro-inflammatory gene expression in macrophages. CSA13 did not reduce the expression of LPS-induced pro-inflammatory genes. (B) The human colonic NCM460 epithelial cells were treated with CSA13 for 24 hours. The cell viability was determined by MTS assay at 490nm. (C) NCM460 cells were grown to >95% confluence. The serum-starved cell culture was scratched by a P200 pipette tip to make a gap. CSA13 (0.3μM) were added to the culture and further incubated for 72 hours. The gap images were taken at 200X magnification. CSA13 promoted gap closure. The results are representative of 3 experiments.

Supplementary Figure 4

Subcutaneous CSA13 administration reduced colonic inflammation in T-cell transfer colitis model.

(A) Experimental plan of T-cell transfer model of colitis. (B) Changes in body weight. (C) H&E staining. (D) Histology score (E) Colonic mRNA expression. (F) Alpha diversity (richness as measured by Chao1) is shown for fecal samples of the mice (4-5 mice per group). Principal coordinates plot of unweighted UniFrac for all mice. The significance of differences in microbial composition (beta diversity) across groups was determined using a permutational method (PERMANOVA), and the p-value is shown in the plot. The abundance of bacteria is shown in bar graphs.

Supplementary Table 1

Cohort 1

Baseline Characteristics

	Non-IBD	UC	CD
FPRL1 mRNA Expression (fold)	1.7±0.31	3.2±0.65	7.1±2.55
Age at Collection (mean ± SEM)	60±2.2	41±2.1	40±2.1
Gender (% Male)	73	55	73
histology score (mean ± SEM)	2.6±0.3	7.5±0.4	8.4±0.4
n	40	50	44

Supplementary Table 2

Cohort 1 - Ulcerative Colitis

Baseline Characteristics

	Low	Middle	High	
Range of FPRL1 mRNA Expression (fold)	0-1.00	1.01-3.30	3.82-106.65	
Age at Collection (mean ± SEM)	43.4±4.36	42.7±3.97	36.6±2.24	NS
Gender (% Male)	47	60	50	
Percentage Who Used Biologics	24	33	14	
Percentage Who Used 6MP or Steroids	71	47	29	
Duration of Disease in Years (mean ± SEM)	16.9±3.26	16.4±4.04	9.6±2.20	NS
CRP Levels (mg/L) (mean ± SEM)	1.2±0.39	3.0±1.05	5.4±1.52	p=0.0098 low vs. high
Simple Clinical Colitis Activity (mean ± SEM)	4.9±0.84	7.3±1.05	7.0±1.09	NS
histology score (mean ± SEM)	6.3±0.63	7.5±0.60	9.4±0.77	p=0.0038 low vs. high
Immune cell subscore (mean ± SEM)	3.3±0.32	4.1±0.34	4.6±0.44	p=0.0001 low vs. high
n	17	16	16	

Cohort 1 - Crohn's Disease

Baseline Characteristics

	Low	Middle	High	
Range of FPRL1 mRNA Expression (fold)	0-1.17	1.7-3.82	4.34-105.15	
Age at Collection (mean ± SEM)	38.75±4.06	41.15±3.80	38.58±4.21	NS
Gender (% Male)	58	77	75	
Percentage Who Used Biologics	31	43	20	
Percentage Who Used 6MP or Steroids	62	43	60	
Duration of Disease in Years (mean ± SEM)	17.90±3.00	16.85±3.23	15.17±3.94	NS
CRP Levels (mg/L) (mean ± SEM)	2.94±1.45	1.73±0.73	2.64±1.02	NS
HBI (mean ± SEM)	5.90±1.10	8.20±1.66	7.00±1.46	NS
histology score (mean ± SEM)	6.92±0.49	8.79±0.89	10.00±0.87	p=0.0014 low vs. high
Immune cell subscore (mean ± SEM)	3.36±0.37	4.43±0.40	5.00±0.58	p=0.0226 low vs. high
Presence of Stricture (%)	27	7	43	
n	15	14	14	

Supplementary Table 3

Cohort 1 - Crohn's Disease

Baseline Characteristics

	below 5 fold	above 5 fold	
Range of FPRL1 mRNA Expression (fold)	0.01-4.87	5.45-105.15	
Age at Collection (mean ± SEM)	39.37±2.62	41.30±4.76	NS
Gender (% Male)	67	80	
Percentage Who Used Biologics	36	32	
Percentage Who Used 6MP or Steroids	50	67	
Duration of Disease in Years (mean ± SEM)	16.44±2.04	16.90±4.72	NS
CRP Levels (mg/L) (mean ± SEM)	2.33±0.74	2.82 ±1.32	NS
HBI (mean ± SEM)	6.82 ±0.91	7.63 ±1.77	NS
histology score (mean ± SEM)	8.12±0.52	9.00±0.96	NS
Immune cell subscore (mean ± SEM)	4.10±0.31	4.33±0.63	NS
Presence of Stricture (%)	16	55	
n	32	11	

Cohort 1 - Crohn's Disease

Baseline Characteristics

	strictured	non-strictured	
Colonic FPRL1 mRNA Expression (fold)	16.60±9.16	3.37±0.86	p=0.0196
Age at Collection (mean ± SEM)	44.45±4.13	45.14±2.53	NS
Gender (% Male)	55	73	
Percentage Who Used Biologics	30	38	
Percentage Who Used 6MP or Steroids	70	46	
Duration of Disease in Years (mean ± SEM)	17.80±4.04	18.00±2.26	NS
CRP Levels (mg/L) (mean ± SEM)	0.57±0.17	3.21±0.81	NS
HBI (mean ± SEM)	4.56±0.43	8.88±1.12	p=0.0316
histology score (mean ± SEM)	8.33±0.87	8.66±0.53	NS
Immune cell subscore (mean ± SEM)	4.22±0.60	4.25±0.31	NS
n	11	32	

Supplementary Table 4

Cohort 2

Baseline Characteristics

	Non-IBD	UC	CD
FPRL1 mRNA Expression (fold)	1.2±0.38	4.9±1.04	5.3±1.59
Age at Collection (mean ± SEM)	53±5.6	41±2.2	37±2.2
Gender (% Male)	54	55	54
n	13	47	36

Cohort 2 - Crohn's Disease

Baseline Characteristics

	FPRL1 below 5 fold	FPRL1 above 5 fold	
Range of FPRL1 mRNA Expression (fold)	0-4.7	5-53.9	
Age at Collection (mean ± SEM)	38.1±2.82	36.1±3.52	NS
Gender (% Male)	42	55	
n	25	11	

Supplementary Figure 1

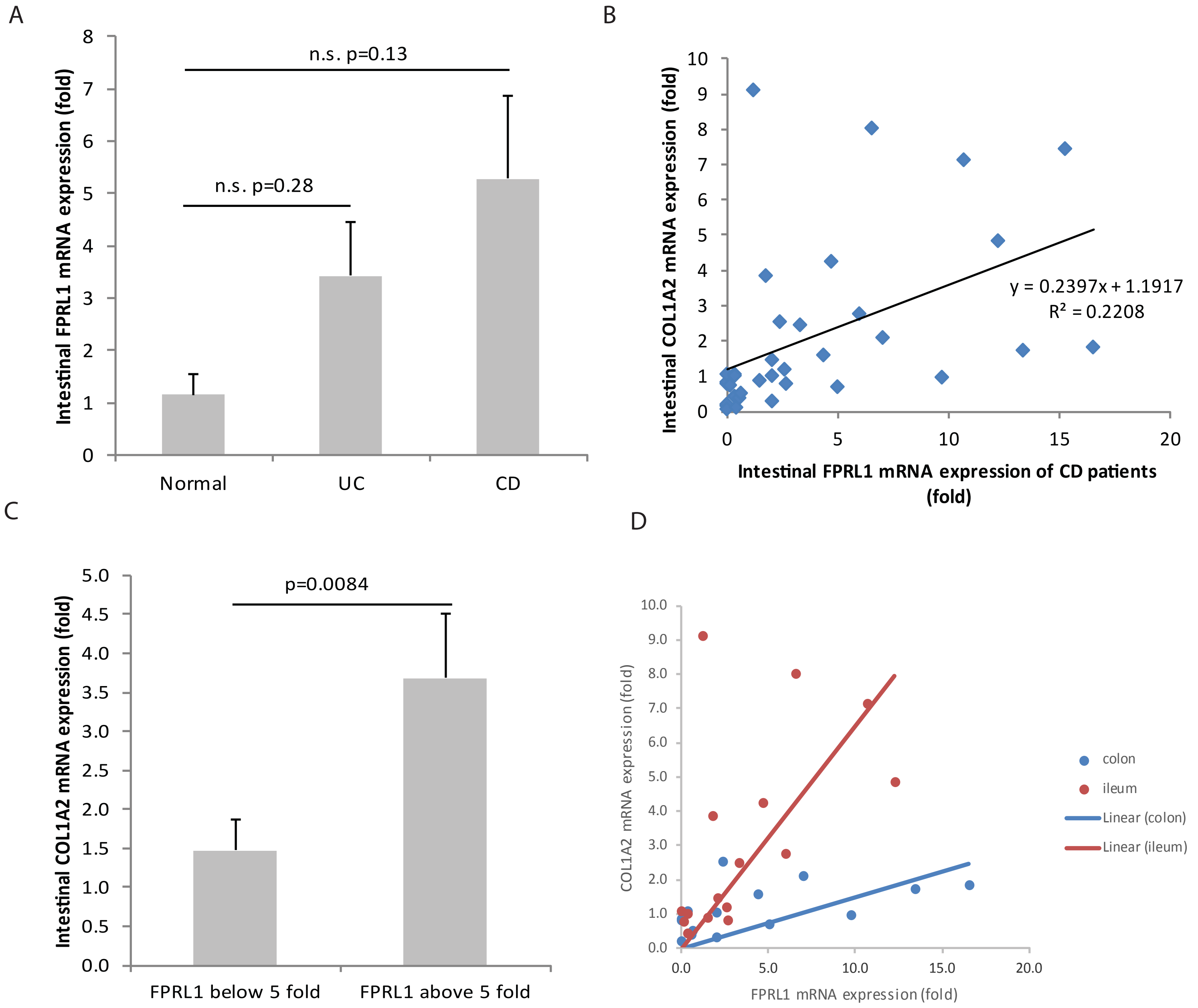

Supplementary Figure 2

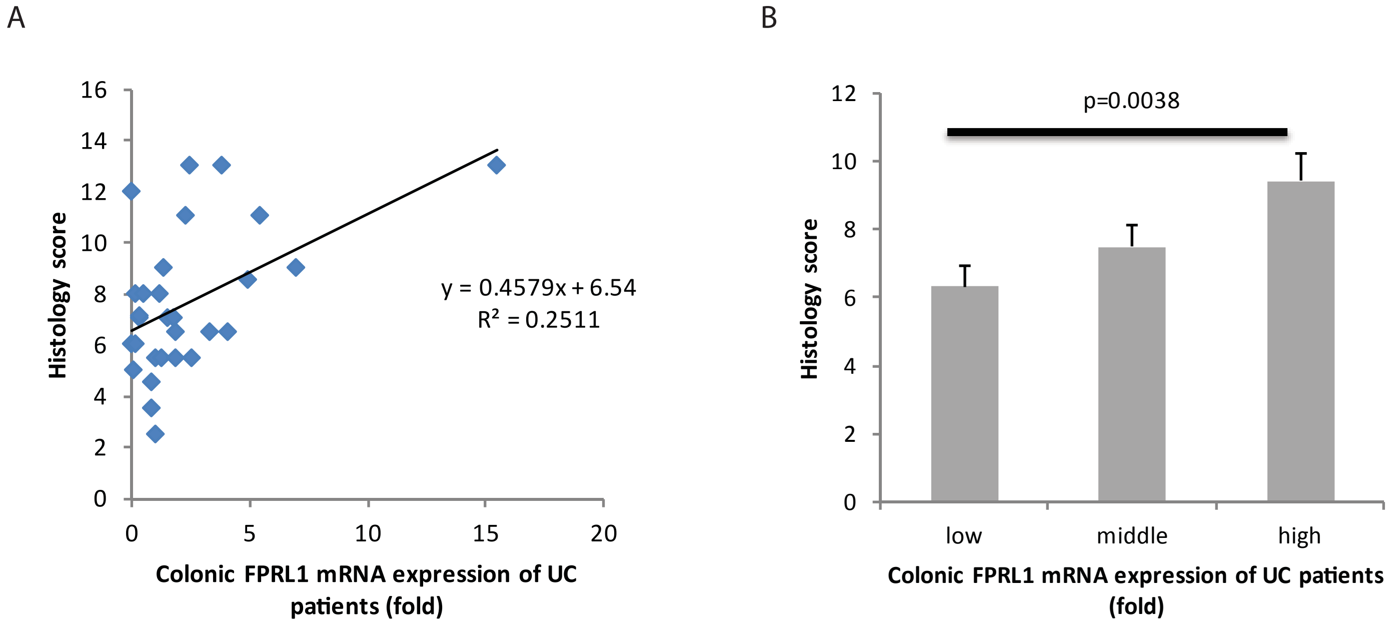

Supplementary Figure 3

A

Relative to untreated control (relative difference in fold)									
average					sem				
Control	CSA13	LPS	LPS + CSA13		Control	CSA13	LPS	LPS + CSA13	
1.00	0.61	0.29	0.53		0.00	0.47	0.14	0.03	Akt1
1.00	1.01	1.43	1.65		0.00	0.25	0.13	0.44	Birc3
1.00	1.35	2.24	3.34		0.00	0.39	0.91	0.23	Camp
1.00	1.11	0.87	1.04		0.00	0.10	0.52	0.07	Card6
1.00	1.16	0.41	0.52		0.00	0.54	0.15	0.07	Card9
1.00	0.70	3.06	3.40		0.00	0.60	0.20	0.74	Casp1
1.00	0.68	1.76	2.25		0.00	0.41	0.00	0.34	Casp8
1.00	1.12	43.76	69.56		0.00	0.20	7.71	29.10	Ccl3
1.00	0.78	23.45	44.72		0.00	0.57	5.34	21.31	Ccl4
1.00	0.61	1.72	2.21		0.00	0.53	0.65	0.02	Cd14
1.00	0.69	0.77	0.97		0.00	0.56	0.08	0.10	Chuk
1.00	2.37	0.18	0.32		0.00	0.24	0.00	0.11	Fadd
1.00	0.85	1.31	1.40		0.00	0.17	0.16	0.08	Hsp90aa1
1.00	1.05	0.56	0.89		0.00	0.04	0.19	0.01	Ikbkb
1.00	0.92	0.29	0.54		0.00	0.08	0.10	0.02	Irak1
1.00	0.94	3.01	3.56		0.00	0.12	1.56	0.94	Irak3
1.00	0.88	0.65	1.52		0.00	0.17	0.23	0.37	Irf5
1.00	0.63	1.86	2.92		0.00	0.60	0.92	0.03	Irf7
1.00	0.95	0.82	1.24		0.00	0.04	0.40	0.19	Jun
1.00	0.70	1.31	1.34		0.00	0.58	0.42	0.34	Ly96
1.00	0.79	0.28	0.50		0.00	0.64	0.01	0.01	Lyz2
1.00	0.81	1.48	1.72		0.00	0.37	0.56	0.02	Map2k1
1.00	1.10	0.55	0.59		0.00	0.19	0.33	0.11	Map2k3
1.00	0.94	1.52	2.21		0.00	0.10	0.36	0.23	Map2k4
1.00	0.98	0.65	0.95		0.00	0.05	0.07	0.12	Map3k7
1.00	1.90	0.56	0.73		0.00	1.49	0.13	0.01	Mapk1
1.00	1.16	0.41	0.48		0.00	0.38	0.12	0.05	Mapk14
1.00	1.93	0.66	0.82		0.00	1.69	0.28	0.09	Mapk3
1.00	1.36	0.66	0.72		0.00	0.64	0.19	0.03	Mapk8
1.00	0.79	0.24	0.46		0.00	0.79	0.00	0.18	Mefv
1.00	0.71	0.97	1.78		0.00	0.53	0.03	0.57	Myd88
1.00	0.94	2.54	4.91		0.00	0.07	0.52	1.44	Nfkb1
1.00	0.69	5.05	6.60		0.00	0.52	0.76	0.46	Nfkbia
1.00	1.16	1.21	1.45		0.00	0.16	0.78	0.59	Nlrc4
1.00	1.03	0.38	0.67		0.00	0.13	0.12	0.01	Nlrp1a
1.00	0.66	3.67	7.26		0.00	0.26	0.80	3.07	Nlrp3
1.00	1.98	11.36	17.72		0.00	0.36	4.35	0.57	Nod1
1.00	0.74	2.39	3.52		0.00	0.52	0.60	0.36	Nod2
1.00	1.63	0.76	0.79		0.00	1.10	0.33	0.21	Pik3ca
1.00	0.95	2.06	2.96		0.00	0.13	0.88	0.20	Pstpip1
1.00	0.86	0.35	0.52		0.00	0.08	0.10	0.03	Pycard
1.00	0.67	0.41	0.66		0.00	0.51	0.01	0.11	Rac1
1.00	0.95	0.59	1.23		0.00	0.17	0.33	0.26	Rela
1.00	0.97	0.50	0.76		0.00	0.03	0.15	0.02	Ripk1
1.00	0.94	3.33	5.37		0.00	0.26	1.34	2.75	Ripk2
1.00	1.03	1.27	1.78		0.00	0.06	0.73	0.33	Slc11a1
1.00	1.23	1.11	1.27		0.00	0.54	0.21	0.04	Sugt1
1.00	2.57	0.92	2.00		0.00	2.95	0.56	0.23	Ticam1
1.00	1.11	0.45	0.56		0.00	0.28	0.18	0.01	Ticam2
1.00	0.88	0.92	1.29		0.00	0.82	0.45	0.04	Tirap
1.00	0.88	2.22	2.84		0.00	0.30	0.30	0.84	Tlr1
1.00	3.70	0.82	1.69		0.00	4.63	0.15	0.63	Tlr2
1.00	0.97	0.66	0.95		0.00	0.14	0.07	0.19	Tlr4
1.00	1.03	1.77	1.80		0.00	0.22	0.39	0.27	Tlr6
1.00	1.02	0.31	0.77		0.00	0.04	0.02	0.39	Tlr9
1.00	0.96	3.14	9.35		0.00	0.03	0.25	4.34	Tnf
1.00	0.72	1.26	2.14		0.00	0.54	0.62	0.08	Tnfrsf1a
1.00	0.80	0.96	1.33		0.00	0.30	0.50	0.22	Tollip
1.00	1.70	1.04	1.27		0.00	1.26	0.46	0.11	Traf6
1.00	3.13	1.08	1.62		0.00	0.01	0.11	0.51	Xiap
1.00	1.16	5.24	5.23		0.00	0.74	1.86	0.47	Zbp1
1.00	0.85	0.46	1.90		0.00	0.05	0.34	0.36	Gusb
1.00	0.91	1.13	1.28		0.00	0.28	0.23	0.04	Hprt
1.00	1.09	0.60	0.80		0.00	0.22	0.23	0.09	Hsp90ab1

Antibacterial response PCR array
Raw264.7 macrophages 4 hours

B

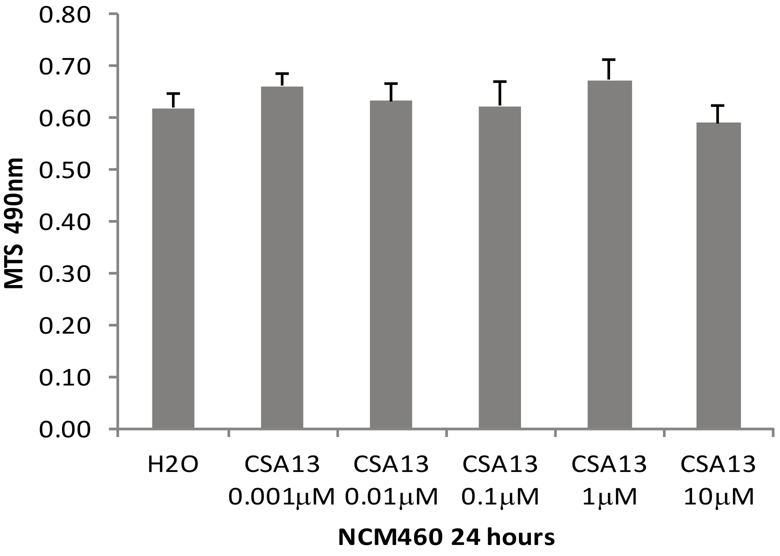

C

H_2O

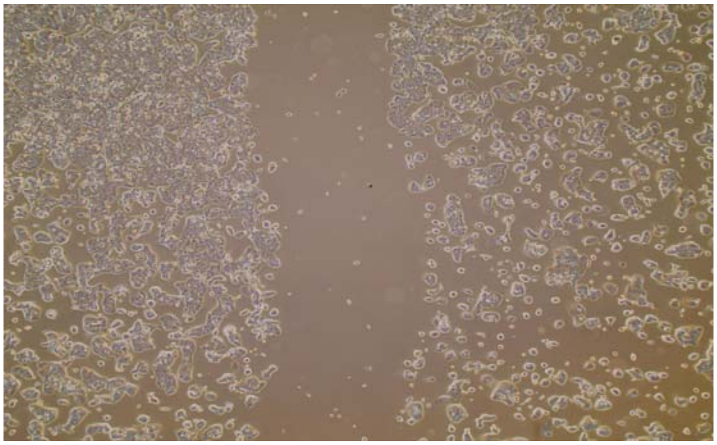

CSA13 0.3μM

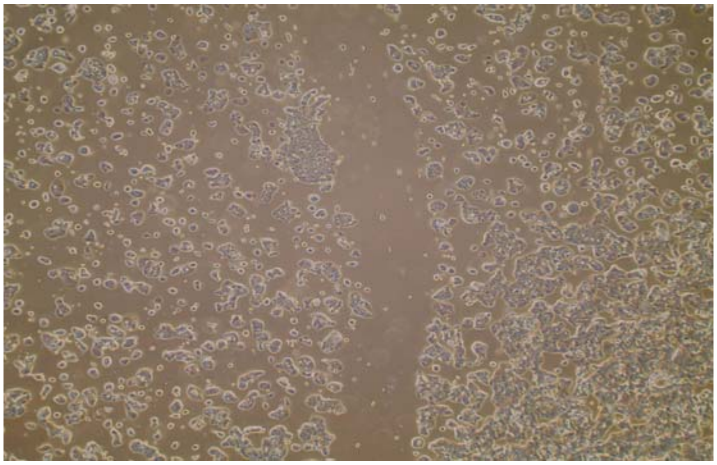

Wound healing assay NCM460 cells 72 hours 200X

Supplementary Figure 4

A

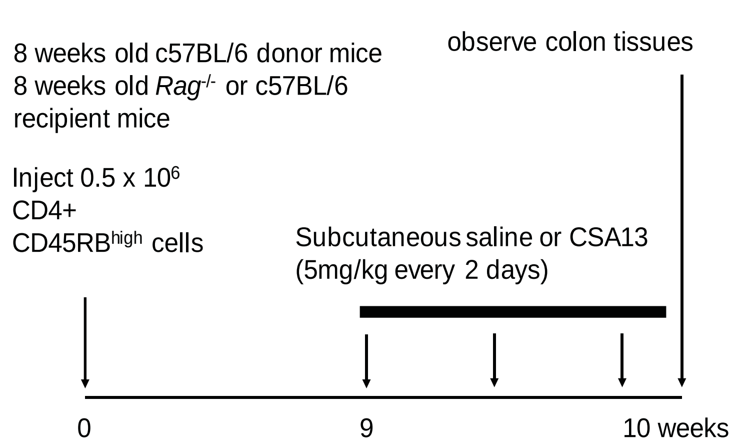

B

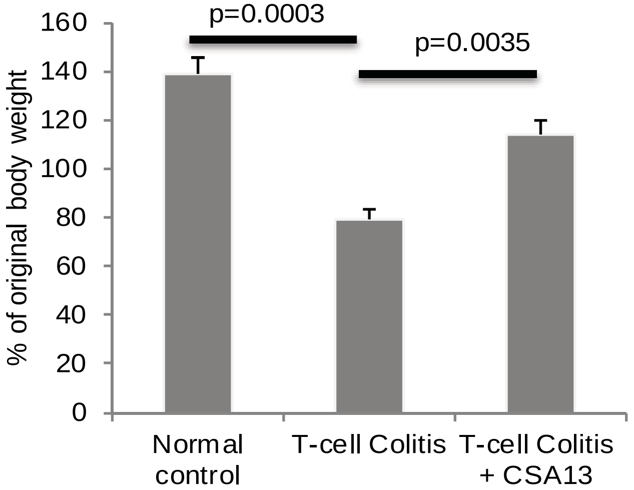

C

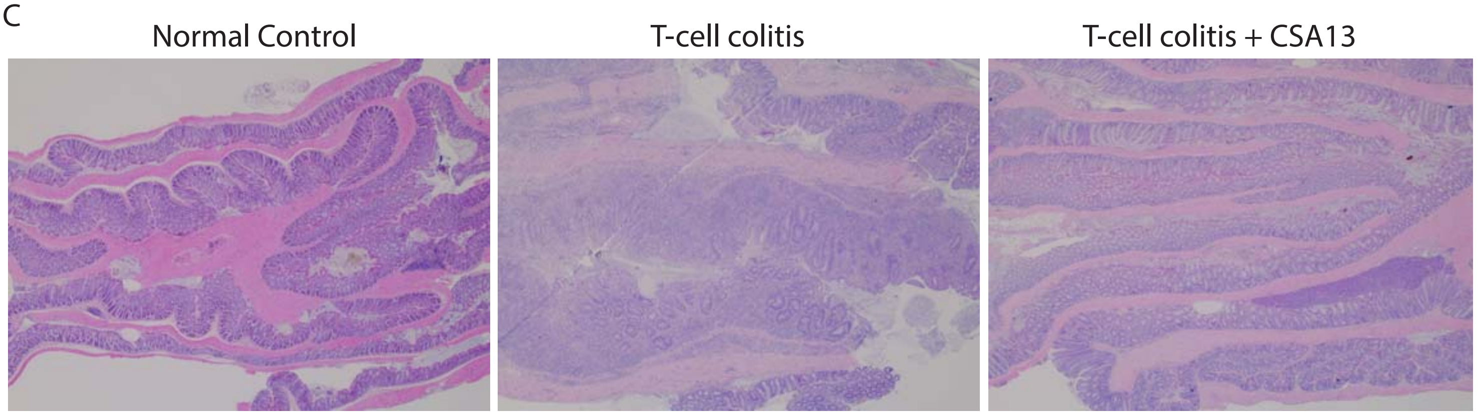

Colonic tissue H&E staining 40X

D

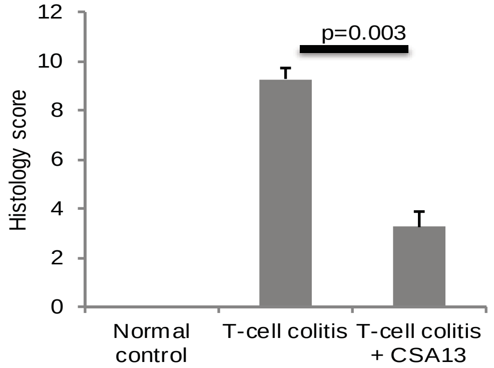

E

Colonic mRNA expression	TNF	F4/80	MPO
Normal control	1.07±0.11	1.05±0.11	1.24±0.23
T-cell colitis	13.82±1.15	2339±377	3.60±0.56
T-cell colitis + CSA13	2.29±0.62	3.37±0.58	1.47±0.18
T-cell colitis vs. control	p=0.0001	p=0.0016	p=0.005
CSA13 vs T-cell colitis	p=0.0001	p=0.0016	p=0.0011

F

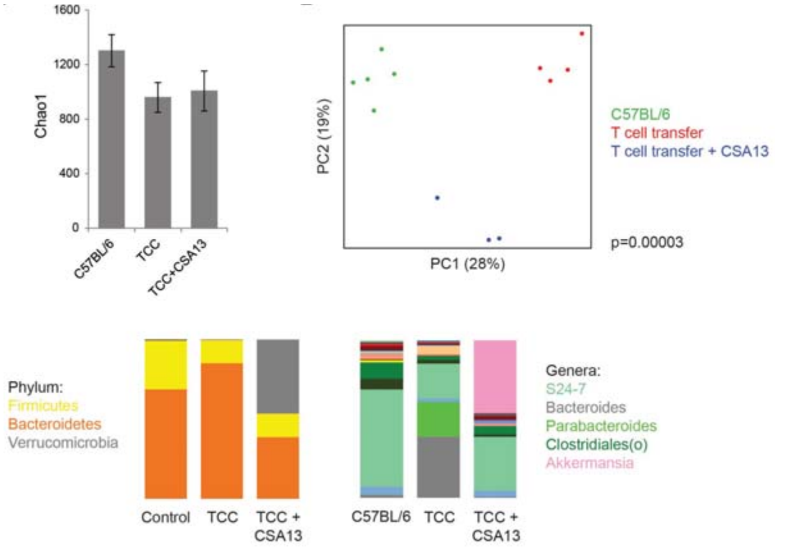

Supplement: Supplementary file 1 — Supplementary Information [file 41598_2017_16753_MOESM1_ESM.pdf]
